# Uterine Tumours Resembling Ovarian Sex-Cord Tumors: A Case Report and Review of the Literature

**DOI:** 10.3390/jcm12227131

**Published:** 2023-11-16

**Authors:** Martina Ferrara, Basilio Pecorino, Maria Gabriella D’Agate, Giuseppe Angelico, Ettore Domenico Capoluongo, Umberto Malapelle, Francesco Pepe, Paolo Scollo, Liliana Mereu

**Affiliations:** 1 Department of Obstetrics and Gynecology, Cannizzaro Hospital, University of Enna “Kore”, 94100 Enna, Italy; eliopek@gmail.com (B.P.); gabrielladagate@gmail.com (M.G.D.); paolo.scollo@unikore.it (P.S.); 2 Department of Anatomic Pathology and Histology, Cannizzaro Hospital, 95100 Catania, Italy; giuangel86@hotmail.it; 3 Department of Clinical Pathology and Genomics, Cannizzaro Hospital, 95100 Catania, Italy; edotto70@gmail.com; 4 Department of Public Health, University of Naples Federico II, 80126 Naples, Italy; umbertomalapelle@gmail.com (U.M.); pepefrancesco88@yahoo.it (F.P.); 5 Department of Obstetrics and Gynecology, Policlinico G Rodolico, CHIRMED, University of Catania, 95124 Catania, Italy

**Keywords:** uterine tumor resembling ovarian sex cord tumor (UTROSCT), review

## Abstract

Uterine tumors resembling ovarian sex-cord tumors (UTROSCT) are thought to develop from pluripotent uterine mesenchymal cells or endometrial stromal cells with secondary sex-cord differentiation. The patient was a 73-year-old postmenopausal woman who had abnormal vaginal bleeding, and she underwent a laparoscopic hysterectomy with bilateral salpingo-oophorectomy. The diagnosis was a case of UTROSCT. A scoping review of the UTROSCT case report present in the literature has been conducted, and 63 articles were found, of which 45 were considered for the 66 clinical cases examined. At the time of diagnosis, six metastatic localizations were found in 59 patients undergoing demolitive surgery (10.2%). Recurrences were diagnosed in 13/59 (22%) patients with multiple locations. A molecular study was performed in 18/66 cases (27.3%) and genetic alterations were found in 10/18 (55.6%) patients. UTROSCTs are considered rare uterine tumors, typically with a favorable prognosis, and are generally considered to have a good prognosis. But, from the review done, they may already manifest themselves at advanced stages, with the possibility of recurrences even at a distance. It would, therefore, be important to be able to define the most aggressive forms and, perhaps, molecular investigation with sequencing could help identify patients most at risk.

## 1. Introduction

Endometrial stromal tumors are not very frequent, and, occasionally, they might be difficult to diagnose. Uterine tumors resembling ovarian sex-cord tumors (UTROSCTs), which are uterine tumors resembling ovarian sex-cord tumors, were initially characterized by Morehead and Bowman in 1945 [1].

Then, based on clinical and histopathologic characteristics, Clement and Scully, in 1976, characterized 14 similar cases and further divided the neoplasms into two separate types [2].

Type I, endometrial stromal tumor with sex-cord-like elements (ESTCLEs) shows a predominant endometrial stromal pattern with areas of sex-cord-like structures that make up approximately 10–40% of the total tumor mass. The tumors are known as endometrial stromal tumors with sex-cord-like components and have a risk of metastasis and recurrence (ESTSCLEs) [3,4]. 

UTROSCTs, or type II uterine tumors resembling ovarian sex-cord tumors, are uncommon and often behave benignly [5]. 

The latest World Health Organization classification of female genital tumors, recognized in 2020, defined UTROSCT as a uterine tumor similar in shape to ovarian sex-cord tumors and further clarified that there is no discernible endometrial stromal component in this tumor tissue [6].

Our clinical case encouraged us to study the literature on this rare tumor to evaluate its behavior and prognosis.

## 2. Material and Method

### 2.1. Study Design

We conducted a scoping review, which allows a broad search while performing a systematic search, even though it does not require methodological appraisal or grading of the evidence [7].

### 2.2. Systematic Database Search

The electronic literature search was conducted from 1996 to November 2022 using PubMed/MEDLINE for English language abstracts. The search included the following medical subject headings (MeSH) or keywords: ‘uterine tumor resembling ovarian sex-cord tumors’, ‘UTROSCT’, and ‘case report’ studies published in English. 

### 2.3. Eligibility Criteria

We included case reports or series, and other descriptive studies regarding the abovementioned research question. Literature reviews and guidelines published by scientific societies were also considered.

### 2.4. Exclusion Criteria

We excluded case reports and case series that did not report personal data, surgery, diagnosis, and immunohistochemistry.

### 2.5. Study Selection

The papers were retrieved by two authors independently; Mendeley was used to store the articles and delete duplicates. The two researchers screened all record titles and abstracts by using PUBMED; those with insufficient information were screened in full text. Disagreement between the reviewers was solved by discussion after reading the full text. The literature search was stopped in November 2022. 

## 3. Case Report

The patient was a 73 year old postmenopausal woman with a history of four pregnancies (two full-term live births; two abortions in the first trimester of pregnancy) who had a history of irregular, abnormal vaginal bleeding for a few days. There was no family history of gynecological cancer. At physical examination, she was found to be in good general health, alert, and pale, with a flaccid abdomen and no signs of peritoneal irritation. At pelvic examination, the uterine volume appeared to be increased by two times the standard volume. The endometrial thickness was recorded at 18 mm by transvaginal ultrasonography and without evidence of uterine masses. Laboratory blood tests showed no significant abnormalities. In May 2022, the patient underwent operative hysteroscopy. Histopathology of the curettings showed endometrial hyperplasia without atypia. The patient, made aware of the risks of the surgery, asked to undergo laparoscopic hysterectomy with bilateral salpingo-oophorectomy. In addition, she received anti-inflammatory, rehydration, and anticoagulation therapy conventionally after surgery. After 28 h postsurgery, no intraoperative and postoperative complications occurred. After follow ups of 12 months and every six months, no recurrence occurred.

### 3.1. Pathological Features 

**Figure 1:**
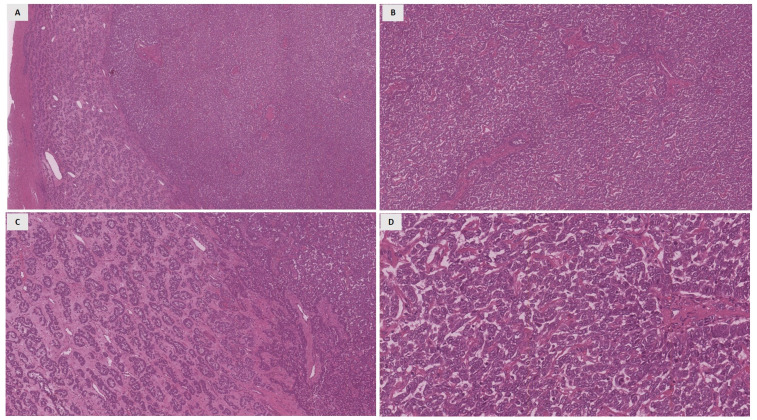
Haematoxylin and eosin-stained sections illustrate the histopathological features of the present case. (**A**): On low power (4×) a hypercellular tumor with pushing margins is observed. (**B**,**C**): On medium power (10×), different patterns of growth were evident; neoplastic cells with diffuse patterns of growth (**B**) or arranged in tubules (**C**). (**D**): on high power (40×), neoplastic cells showed epithelioid morphology, with round, slightly irregular nuclei, and scant eosinophilic cytoplasm without evidence of nuclear atypia or mitotic figure.

Macroscopic examination showed multiple nodule lesions within the myometrium whose diameters ranged between 0.5 to 6 cm. All nodules except one showed macroscopic and microscopic features consistent with leiomyomas. On the other hand, a nodular lesion, measuring 2.5 cm in its greatest diameter, showed a yellowish cut surface and a solid consistency. Histologically, the neoplasia showed a diffuse pattern of growth with an alternating cord-like pattern and tubular and trabecular areas. (Figure 1A–C). Only focally large trabeculae were observed. Neoplastic cells were small in size and showed epithelioid morphology, with round, slightly irregular nuclei and scant eosinophilic cytoplasm (Figure 1D). Morphological features, such as necrosis, mitotic activity, lymphovascular invasion, and infiltrative margins, were not observed.

**Figure 2:**
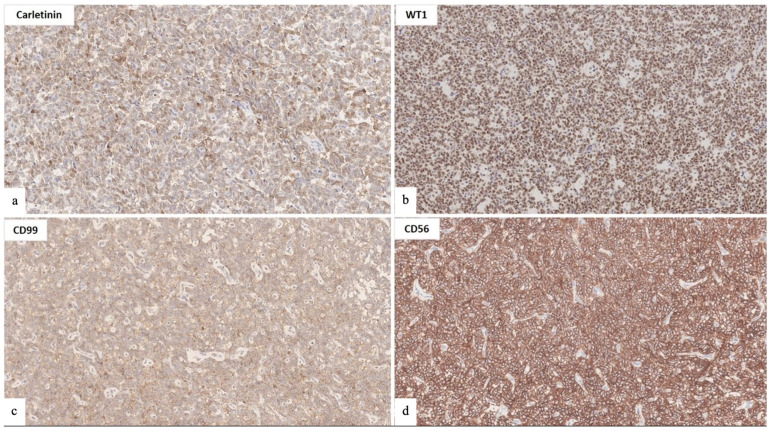
Immunohistochemical stains for markers of sex-cord differentiation: (**a**): Carletinin; (**b**): WT1; (**c**): CD99; and (**d**): CD56.

Through immunohistochemistry, neoplastic cells showed immunoreactivity for mesenchymal (vimentin, desmin) and epithelial (pan-cytokeratin AE1/AE3) markers. Estrogen and progesterone receptors were also positive. Moreover, the immunoreactivity for markers of sex-cord differentiation, including calretinin, CD99, CD56, and WT1 was also observed (Figure 2a–d). On the other hand, neoplastic cells were negative for smooth muscle actin, caldesmon, HMB45, Melan-A, and CD10. Based on the abovementioned morphological and immunohistochemical findings, the diagnosis of a uterine tumor resembling an ovarian sex-cord tumor (UTROSCT) was rendered.

### 3.2. Tissue-Sample Management

Overall, a series of four slides (5 microns) and a matching hematoxylin and eosin (H&E)-stained section was assessed for molecular analysis. The tumor tissue was manually microdissected by adopting a sterile blade and incubated overnight (O.N) at 56 °C with proteinase K. After this, nucleic acids were purified following the manufacturer instructions of the AllPrep DNA/RNA Kit (Qiagen, Hilden, Germany) [8]. Briefly, genomic RNA (gRNA) was recovered on a proprietary filter column and eluted in 30 µL of DNAse and RNAse-free water (Thermo Fisher Scientifics, Waltham, MA, USA) in accordance with standardized procedures [8]. Finally, gRNA was evaluated on the TapeStation 4200 microfluidic platform adopting a dedicated ScreenTape device (Agilent Technologies, Santa Clara, CA, USA). This system enables the calculation of the RNA concentration (pg/µL) and the RNA Integrity Number (RIN), a measurement of RNA fragmentation. The sample was stored at −20 °C until molecular analysis [9].

### 3.3. NGS Analysis

Molecular analysis was carried out by adopting an Oncomine Precision Assay (OPA) panel on a fully automatized Genexus platform (Thermofisher Scientifics) following manufacturer procedures. Briefly, this platform allows for the automatic analysis of DNA/RNA samples (from library preparation to data interpretation) within 24 h. The OPA assay covers 50 cancer-related actionable genes, including the most common intergenic fusions in n = 16 actionable genes (*ALK*, *ROS1*, *NTRK1-3*, *RET*, *FGFR1-3*, *NRG1*, *RSPO2-3*, *NUTM*, *ESR1*, *BRAF*, and *NRG1*). Briefly, a sample sheet was generated on a dedicated server and assigned to a new run. The NGS platform was manually loaded with OPA primers, strip solutions, strip reagents, and supplies according to the manufacturer’s instructions. A total of 10 ng was dispensed into a 96-well plate and put on the Genexus platform. Finally, a sequence analysis was carried out on the GX5TM chip that allows for simultaneous processing of n = 8 samples in a single line by adopting an OPA assay. Data analysis was performed on the proprietary Genexus software, as recommended by the manufacturer’s guidelines. Particularly, detected alterations were annotated by adopting Oncomine Knowledgebase Reporter Software (Oncomine Reporter 5.0) [10,11]. A microfluidic analysis highlighted an RNA concentration of 858.0 pg/µL. Moreover, fragmentation analysis revealed a DIN of 2.8. From a technical point of view, a median number of 1,215,781.0 total reads, 59,296.0 mapped reads, and 95 mean read lengths were identified, respectively. No clinically relevant aberrant transcripts in cancer-related genes covered by the OPA assay were detected (Table 1).

## 4. Results

**Figure 3:**
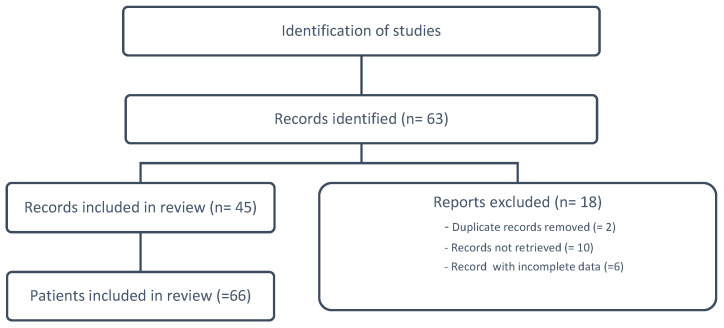
Flow diagram for scoping reviews, which included searches of PUBMED.

After the search that included the following medical subject headings (MeSH) or keywords ‘uterine tumor resembling ovarian sex-cord tumors’, ‘UTROSCT’, and ‘case report’ of studies published in English was completed, 63 articles were found, of which 45 were considered for a total of 66 clinical cases examined (Figure 3). The 66 patients examined were aged between 22 and 77 years old (average age 49.7 years). All patients underwent immunohistochemical investigation, which contributed to the differential diagnosis, validating the diagnosis of UTROSCT. For 4/66 (6%) patients, a follow up is not reported. The clinical cases that have been subjected to molecular investigation are 14/66 (21.2%). Thirty-seven out of 66 patients (56.1%) underwent subtotal/total hysterectomy with bilateral salpigo-oophorectomy (H-BSO), 10/66 (15.1%) underwent subtotal/total hysterectomy with bilateral salpingectomy (H-BS), and 12/66 (18.2%) underwent total hysterectomy with bilateral salpingo-oophorectomy and removal of pelvic and/or aortic lymph nodes. Finally, 7 patients underwent conservative surgery, of which 5/66 (7.6%) underwent hysteroscopy and 2/66 (3%) underwent myomectomy. At the time of diagnosis, six metastatic localizations were found in 59 patients undergoing demolitive surgery (10.2%). In particular, the following were highlighted: 2/12 (16.7%) localizations of lymph node metastases in patients subjected to the removal of pelvic and/or aortic lymph nodes, 2/49 (4.1%) localizations of ovarian metastases in patients subjected to HBSO/HBSO + lymphadenectomy, and 2/59 (3.4%) localizations of cervical metastases in patients undergoing total hysterectomy. Follow-up was performed in 59/66 patients. The mean duration of follow up was 45.9 months (1–384 months). Two out of seven patients undergoing conservative surgery had no follow up, and none of the patients treated with conservative surgery had a recurrence. Clinical cases treated with demolitive surgery were 59/66 (89.4%); of these, 5/59 (8.5%) did not perform a follow up. Recurrences were diagnosed in 13/59 (22%) patients with multiple locations. Local recurrences involved the ovary, vaginal vault, and pelvic peritoneum. Distant recurrences were localized in the peritoneum, liver, intestine, lung, and lymph nodes (Table 2).

The molecular analysis was performed in 18/66 cases (27.3%). Eight of 18 patients (44.4%) showed no genetic alterations. Genetic alterations were found in 10/18 (55.6%) patients, such as 3/18 (16.7%) fusion of the GREB–NCOA1/2 genes, 3/18 (16.7%) fusion of the ERS1–NCOA2 genes, 2/18 (11%) fusion of the JAZF1–SUZ12 genes, 1/18 (5.6%) GREB1–CTNNB1 fusion transcript detected, and 1/18 (5.6%) translocations of t(X;6)(p22.3;q23.1) and t(4;18)(q21.1;q21.3). Five out of ten (50%) patients with genetic alteration developed disease recurrence and 1/10 (10%) patients had a metastasis at the time of diagnosis.

## 5. Discussion

UTROSCT is a relatively rare disease. About 70 cases of UTROSCT have been described in the literature. The tumor typically affects postmenopausal women and women of childbearing age. The age of onset ranges from 20 to 86 years; the median age is 51 years. The most common symptom is abnormal uterine bleeding or pelvic pain, but it can occur asymptomatically [6,7]. The tumor size ranges from 4 mm to 135 mm (on average 47.6 mm). This type of uterine tumor cannot be suspected using any of the instrumental tests we use (US, CT, or MRI), the precise diagnosis is established with tissue biopsy. According to histology, the tumors are made up of nests that resemble sex cords and epithelioid cells [4].

Studies have revealed that most UTROSCTs are positive for at least two sex-cord-labeled antibodies (CD99, calretinin, melan A, and inhibin), which are frequently joined by smooth muscle (SMA, desmin, and calponin), endometrial stromal (CD10), and other antibodies (vimentin, ER, and PR) with varying degrees of expression. This is true even though immunohistochemical markers did not reveal any specific targets [55].

Taking into consideration their rarity, only a few studies have explored the molecular alterations of UTROSCT. Mutations frequently occurring in ovarian sex-cord tumors, such as *FOXL2* or *DICER1*, have not been observed in UTROSCT. Moreover, *JAZF1–SUZ12* gene fusion, usually observed in endometrial stromal neoplasms has not been described in UTROSCT [3].

However, recent studies demonstrated that the majority of UTROSCTs carry recurrent *NCOA2/3* gene fusions previously discovered in Mullerian adenosarcoma and undifferentiated uterine sarcoma. UTROSCT-harboring *NCOA2/3* gene fusions have been shown to occur mainly in premenopausal patients and show unequivocal morphological and immunohistochemical evidence of sex-cord differentiation [56].

Moreover, rare UTROSCT cases showing fusions involving the *GREB1* gene have also been reported. These latter cases have been shown to occur in older women and may show local recurrences [57].

Despite the fact that the majority of the UTROSCTs behave benignly, they are typically regarded as tumors with low malignant potential [6]. The most frequent treatment pattern (65.1%) was total hysterectomy with bilateral adnexectomy, followed by total hysterectomy alone (18.6%), and mass resection alone (14%), respectively [58].

However, the uncertain behavior of the UTROSCTs emerged from this scoping review. In fact, we found that, in 10.2% of cases, metastases were found at the time of diagnosis. Furthermore, 22% of the cases in the literature with long-term follow up found the onset of recurrences with local localization (ovarian, vaginal, and pelvic) or metastasis (peritoneal, hepatic, lymph node, and pulmonary). This highlights the behavior of a pathology with an uncertain course, which deserves greater attention. To this end, on the basis of this scoping review, we underline the need to undertake an individualized treatment on the patient that can take into consideration the communication to the same of this pathology and, therefore, propose demolitive surgery in the first instance. If the patient wishes to perform conservative treatment, she must be informed of the risks of recurrence and the need to carry out close follow ups. To this must be added the importance of adding genetic investigation to the histological and molecular investigation, which can guide the choice of the clinician for the purpose of better management of the clinical case.

## 6. Conclusions

Until now, UTROSCTs, according to the WHO, are benign in most cases but should be considered to have low malignant potential because they may recur. Hysterectomy and mass resection alone are potential therapeutic options if risk indicators for recurrence, like genetic alteration, are not present. We, therefore, suggest considering this pathology to have uncertain behavior, carefully counseling the patient, and showing the risks of metastasis and recurrences, and, therefore, also personalizing treatment on the basis of molecular and genetic investigations.

## Figures and Tables

**Table 1:** Scoping review of case report and case series of literature. H + BSO: hysterectomy + bilateral salpingo-oophorectomy; H + BS: hysterectomy + bilateral salpingectomy; H + BSO + LND: hysterectomy, bilateral salpingo-oophorectomy, and pelvic and para-aortic lymph node dissection; F-U: follow up; RT: radiotherapy; CHT: chemotherapy.

N.	Article, Year	Patient	Risk Factor	Surgery	Metastasis	Immunohistochemical Antibodies	Gene Fusion and Somatic Mutation Analyses	Diagnosis	Adjuvant Therapy	Follow Up	Recurrence
1	Abdullazade et al., 2010 [12] Case series	Case 1: 46 year old Case 2: 30 year old Case 3: 42 year old	Case 1: - Case 2: - Case 3: -	Case 1: Hysterectomy, bilateral salpingo-oophorectomy Case 2: Myomectomy Case 3: Hysterectomy, bilateral salpingo-oophorectomy	Case 1: - Case 2: - Case 3: -	Case 1: CD56+, Calretinin+, Inhibin,+ Desmin+, AE1/AE3+ Case 2: Inhibin+ Case 3: CD56, Calretinin+, Inhibin+, Desmin+, AE1/AE+	Not Performed	Case 1, 3: UTROSCT	Case 1, 3: -	Case 1: 24 months Case 2: Not revealed Case 3: Lost in follow-up	Case 1: - Case 2: Not revealed Case 3: Not revealed
2	Bennett et al., Nov. 2020 [13] Case series	Case 1: 37 year old Case 2: 54 year old Case 3: 30 year old	Case 1: - Case 2: - Case 3: -	Case 1: Hysterectomy with bilateral salpingectomy Case 2: Supracervical Hysterectomy with bilateral salpingo-oophorectomy Case 3: Hysterectomy with bilateral salpingectomy	-	Case 1, 2: WT1+ CAM5.2 + Estrogen receptor (ER) + Progesterone receptor (PR) + CD56 + AE1/AE3 + Case 3: WT1 + CAM5.2 + Estrogen receptor (ER) + Progesterone receptor (PR) + CD56 + Calretinin + Desmin + Melan-A + CD10 +	Case 1, 2, 3: ESR1–NCOA2 fusion detected	Case 1, 2, 3: UTROSCT	Case 1: - Case 2: - Case 3: -	Case 1: 84 months Case 2: 108 months Case 3: 384 months	Case 1: Left pelvic sidewall recurrence Case 2: Pelvic recurrence Case 3: Omental recurrence
3	Berretta et al., May 2009 [14] Case report	1, 26 year old	-	Operative Hysteroscopy	-	Desmin + Actin SM/calponin + α-inhibin + Calretinin + Progesteron receptor (PR) + CD99 + WT-1 + Ki-67 increased	Not Performed	UTROSCT	-	Not Revealed	Not Revealed
4	Biermann et al., Jan 2008 [15] Case report	1, 68 year old	-	Hysterectomy and bilateral salpingo-oophorectomy	-	Inhibin + CD99 + CD56 + Pancytokeratin + Cytokeratin 18 + Vimentin + Calretinin + Progesterone receptor (PR) + Estrogen receptor (ER) +	No fusion transcript detected	UTROSCT	-	48 months	Small Bowel
5	Chang et al., 2020 [16] Case report	1, 57 year old	-	Hysterectomy and bilateral salpingo-oophorectomy	-	Progesterone receptor + Estrogen receptor + Desmin + WT-1 + CD56 + CD99 + Ki-67 increased	GREB1–NCOA2 fusion gene	UTROSCT	-	30 months	Pelvic nodule 6.0 × 5.0 cm treated with chemotherapy (3 cycles of paclitaxel liposome and carboplatin)
6	Croce et al., Jan. 2019 [17] Case report	1, 70 year old	-	Hysterectomy and bilateral salpingo-oophorectomy	-	Estrogen receptor (ER) + Progesterone receptor (PR) + CD10 + Desmin + AE1-AE3 + EMA + CK8/18 + Calretinin + WT-1 + Melan A +	GREB1–CTNNB1 fusion transcript detected	UTROSCT	(I Time) - (II Time) Aromatase inhibitors	(I Time) 17 months (II Time) 12 months	(I Time) Widespread pelvic nodule (II Time) Lung metastases and abdominal peritoneal recurrence
7	Czernobilsky et al., 2005 [18] Case report	1, 63 year old	-	Hysterectomy, bilateral salpingo-oophorectomy	-	AE1/AE3 + CK18 + Inhibin + Vimentin + Calretinin + Progesterone Receptor (PR)+	Not Performed	UTROSCT	-	13 months	-
8	Dubruc et al., Feb. 2019 [19] Case report	1, 56 year old	-	Hysterectomy and bilateral salpingo-oophorectomy	-	CKAE1/AE + Smooth muscle actin + Desmin + Calretinin + Inhibin + MelanA + CD99 + CD56 + WT1 +	Not Performed	UTROSCT	-	4 months	-
9	Ehdaivand et al., Jul. 2014 [20] Case report	1, 47 year old	-	Hysterectomy, bilateral salpingo-oophorectomy	-	Not Revealed	Not Performed	UTROSCT	-	24 months	-
10	Garcia et al., Jul. 2018 [21] Case report	1, 46 year old	-	(I Surgery) Vaginal myomectomy (II Surgery after diagnosis) Hysterectomy and bilateral salpingo-oophorectomy	-	CD56 + Smooth muscle actin + CD10 + Desmin + Pan-cytokeratin +	Not Performed	UTROSCT	-	60 months	-
11	Garuti et al., Dec. 2008 [22] Case report	1, 27 year old	-	Operative Hysteroscopy	-	CD99 + α-inhibin + Calretinin + Cytokeratin + Estrogen receptor (ER)+	Not Performed	UTROSCT	-	13 months	-
12	Gill et al., 2021 [23] Case report	1, 46 year old	APC gene positive	Hysterectomy, bilateral salpingo-oophorectomy, omentectomy, bilateral pelvic sentinel lymph node biopsy + Proctocolectomy and transduodenal ampullectomy	-	β-catenin+ Cyclin D1+ Bcl2+ CD10+ Estrogen receptor (ER)+	Not Performed	UTROSCT + Endometrial endometrioid adenocarcinoma G1 (FIGO Stage II) + Adenocarcinoma of the colon	Adjuvant Radiotherapy	Not Revealed	-
13	Giordano et al., Sep. 2010 [24] Case series	Case 1: 26 year old Case 2: 46 year old	Case 1, 2: -	Case 1: Operative Hysteroscopy Case 2: Hysterectomy, bilateral salpingo-oophorectomy	Case 1: - Case 2: cervix	Case 1: Calretinin +, CD99+, α-actin+, Cytokeratin+ Case 2: Calretinin+, CD10+, EMA+, Cytokeratin+	Not Performed	Case 1, 2: UTROSCT	Case 1, 2: -	Case 1: 15 months Case 2: Not revealed	Case 1, 2: -
14	Gomes et al., Nov. 2015 [25] Case report	1, 53 year old	-	(I Surgery) Supracervical hysterectomy (II Surgery after diagnosis) Bilateral salpingo-oophorectomy, omentectomy, parametrectomy, pelvic lymphadenectomy, and uterine cervical resection	Cervix, right parametrium, and right ovarium hilum	Vimentin + CD99 + AE1/AE3 + Estrogen receptor (ER) + Progesterone receptor (PR) + WT-1 + CD10 + Melan-A + Inhibin + Desmin +	Not Performed	UTROSCT	4 cycles of adjuvant-modified BEP (bleomycin + cisplatin+ etoposide)	60 months	-
15	Grither et al., Sept 2020 [26] Case report	1, 69 year old	-	Hysterectomy and bilateral salpingo-oophorectomy	-	CD10 + Progesterone receptor (PR) + Calretinin + Vimentin + Estrogen receptor (ER) + Inhibin +	Somatic GREB1–NCOA1 fusion	UTROSCT	-	8 months	-
16	Hashmi et al. 2014 [27] Case report	1, 48 year old	-	Hysterectomy and bilateral salpingo-oophorectomy	-	Vimentin + CD99 + S100 + Pancytokeratin immunostain + Desmin +	Not Performed	UTROSCT	-	Not Revealed	Not Revealed
17	Hauptmann et al., May. 2001 [28] Case report	1, 49 year old	-	Hysterectomy and bilateral salpingectomy	-	Vimentin + Pancytokeratin + EMA + CD99 + Smooth-muscle + Actin + MIB-1 + Progesterone receptors +	Not Performed	UTROSCT	-	-	-
18	Jeong et al., May 2015 [29] Case report	1, 32 year old	-	(I Surgery) Operative Hysteroscopy (II Surgery after diagnosis) Hysterectomy with bilateral salpingectomy	-	Calretinin + CD99 + CD56 +	Not Performed	UTROSCT	-	47 months	-
19	Kabbani et al., 2003 [30] Case report	1, 24 year old	-	Hysterectomy, bilateral oophoropexy, and pelvic lymph node sampling after radiotherapy and brachytherapy	-	Calretinin + Desmin + CK7 + SMA + Cytokeratins +	Not Performed	UTROSCT	-	12 months	-
20	Kaur et al., 2020 [31] Case series	Case 1: 49 year old Case 2: 42 year old Case 3: 47 year old Case 4: 43 year old Case 5: 46 year old Case 6: 59 year old	Case 1–5: - Case 6: Tamoxifen	Case 1: Radical Hysterectomy Type III and bilateral salpingo-oophorectomy Case 2–6: Hysterectomy and bilateral salpingo-oophorectomy	Case 1, 6: -	Case 1: MIC2 +, Calretinin +, CK +, EMA+, Vimentin+, SMA,+ Estrogen receptor (ER)+, Progesterone receptor (ER)+ Case 2: MIC2+, Calretinin+, CK+, Vimentin+, SMA+, Estrogen receptor (ER)+, Progesterone receptor (ER)+ Case 3: CK,+ Inhibin+, Desmin+, SMA+, Estrogen receptor (ER)+, Progesterone receptor (ER)+ Case 4: CK+, Vimentin+, Desmin+, SMA+, Estrogen receptor (ER)+, Progesterone receptor (ER)+ Case 5: CK+, Desmin+, SMA+, Estrogen receptor (ER)+, Progesterone receptor (ER)+ Case 6: CK+, Desmin+, Estrogen receptor (ER)+ Progesterone receptor (ER)+	Not Performed	Case 1, 6: UTROSCT	Case 1, 2, 4, 5, 6: - Case 3: Carboplatin + Paclitaxel	Case 1: 24 months Case 2: 18 months Case 3: 7 months Case 4: 12 months Case 5: 1 months Case 6: Not revealed	Case 1: - Case 2: - Case 3: Pelvic Recurrence and LND metastasis Case 4: - Case 5: - Case 6: -
21	Khalifa et al., 1996 [32] Case report	28 year old	-	Hysterectomy, bilateral salpingectomy	-	Vimentin+ SMA+ Desmin+ Progesterone receptor (PR)+	-	UTROSCT	-	204 months	Right ovary, omentum, small bowel, sigmoid colon
22	Kimyon Comert et al., Apr. 2018 [33] Case report	1, 61 year old	-	Hysterectomy and bilateral salpingo-oophorectomy	-	CD56 + Vimentin + Calretinin + Progesterone receptor (PR) + Estrogen receptor (ER) + Synaptophysin + Chromogranin +	Not Performed	UTROSCT	-	60 months	Pelvic Mass
23	Kondo et al., Jul. 2017 [34] Case report	1, 69 year old	-	Hysterectomy and bilateral salpingo-oophorectomy	-	CD10 + Progesterone receptor + Estrogen receptor + CD56 + WT1 + Desmin + Vimentin +	Not Performed	UTROSCT	-	36 months	Lung metastasis
24	Kuznicki et al., Sept. 2017 [35] Case report	1, 49 year old	-	Hysterectomy and bilateral salpingo-oophorectomy + Pelvic and para-aortic lymphadenectomy and total omentectomy	Bilateral ovarian surfaces and omentum	CK7 + Vimentin + WT1 + CK20 +	Not Performed	UTROSCT	Postponed due to severe complications	-	Hepatic and peritoneal implants and disease progression in the pelvis
25	Macak et al., 2014 [36] Case report	1, 53 year old	-	Hysterectomy and bilateral salpingo-oophorectomy + Pelvic and paraaortic lymphadenectomy	1 internal iliac artery lymph node	Desmin + Calponin + WT1 + Ki-67 increased	No fusion transcript detected	UTROSCT	-	10 months	-
26	Marrucci et al., Nov. 2019 [37] Case report	1, 73 year old	-	Hysterectomy and bilateral salpingo-oophorectomy	-	Vimentin + CD56 + CD99 + WT1 +	Not Performed	UTROSCT	-	59 months	Vaginal vault
27	Richmond et al., Dec. 2016 [38] Case report	1, 56 year old	-	Hysterectomy, bilateral salpingo-oophorectomy, pelvic and paraaortic lymphadenectomy, omentectomy, and abdominopelvic washings	-	Estrogen receptor (ER) + Progesterone receptor (PR) + CD10 + Vimentin + CD56 + Calretinin + Inhibin + CK20 + CDX2 + CK7 +	No fusion transcript detected	UTROSCT	-	Not revealed	-
28	Sadeh et al., 2017 [39] Case report	1, 57 year old		Hysterectomy and bilateral salpingo-oophorectomy	-	Calretinin + MART-1 + Inhibin + CD99 + Desmin + Actin + Vimentin + Pankeratin +	Not Performed	UTROSCT	-	36 months	-
29	Sato et al., Mar. 2020 [40] Case report	1, 57 year old	-	(Surgery) Hysterectomy and bilateral salpingo-oophorectomy + (II Surgery after diagnosis) Pelvic and para-aortic lymphadenectomy and subtotal omentectomy	-	Calretinin + A-inhibin + CD99 + AE1/AE3 + CD10 + Estrogen receptors + Progesterone receptors +	Not Performed	UTROSCT with sarcomatous features	-	39 months	-
30	Schraag et al., Jan. 2017 [41] Case series	Case 1: 24 year old Case 2: 28 year old Case 3: 72 year old	Case 1: - Case 2: - Case 3: -	Case 1: Two Hysteroscopy and Abdominal Myomectomy Case 2: (I Surgery) Abdominal Myomectomy (II Surgery after diagnosis) Hysterectomy and bilateral salpingectomy Case 3: Hysterectomy and bilateral salpingo-oophorectomy	Case 1: - Case 2: - Case 3: -	Case 1: Calretinin + WT1 + AE1/AE3 + Alpha-SMA + Case 2: Calretinin + WT1 + AE1/AE3 + Alpha-SMA + Case 3: Calretinin + AE1/AE3 + Alpha-SMA + Inhibin +	Case 1, 2, 3: Not Performed	Case 1: UTROSCT Case 2: UTROSCT Case 3: UTROSCT	Case 1: - Case 2: - Case 3: -	Case 1: 56 months Case 2: 20 months Case 3: 46 months	Case 1: - Case 2: Pelvic mass and peritoneal carcinomatosis Case 3: -
31	Segala et al., Jan. 2019 [42] Case report	1, 63 year old	Tamoxifen for bilateral breast carcinoma	Hysterectomy and bilateral salpingo-oophorectomy	-	Vimentin + Smooth-muscle + Actin + EMA + Estrogen receptors + Progesterone receptors +	Not Performed	UTROSCT	-	56 months	-
32	Shibahara et al., Mar. 2022 [43] Case report	1, 77 year old	-	Hysterectomy and bilateral salpingo-oophorectomy	-	Alpha-SMA + Calretinin + CD99 + WT-1 + Estrogen receptor (ER) + Progesterone receptor (PR) + Desmin + H-caldesmon + CAM5.2 + Inhibin +	Not Performed	UTROSCT	-	12 months	-
33	Sitic et al., Mar. 2007 [44] Case report	1, 76 year old	-	Hysterectomy, bilateral salpingo-oophorectomy	-	Vimentin+, CD10+, CD99+, α-actin+	No fusion transcript detected	UTROSCT	-	48 months	-
34	Stolnicu et al., Apr. 2009 [45] Case series	Case 1: 71 year old Case 2: 64 year old	Case 1: - Case 2: Tamoxifen	Case 1: Hysterectomy, bilateral salpingo-oophorectomy Case 2: Hysterectomy, bilateral salpingo-oophorectomy	-	Case 1, 2: CAM 5.2 + CD56 + α-inhibin + Calretinin + CD10 +	Not Performed	Case 1, 2: UTROSCT	-	Case 1: 60 months Case 2: 36 months	Case 1: - Case 2: -
35	Suzuki et al., Oct. 2001 [46] Case report	1, 66 year old	-	Hysterectomy, bilateral salpingo-oophorectomy, partial omentectomy, and pelvic lymphadenectomy	-	CD99 + Keratin + Vimentin +	Not Performed	UTROSCT	-	10 months	-
36	Tatar et al., Jan. 2016 [47] Case report	45 year old	-	Hysterectomy, bilateral salpingo-oophorectomy, and pelvic and para-aortic lymph node dissection	-	C-Kit+, Inhibin+	Not Performed	UTROSCT	-	36 months	-
37	Uçar et al., Dec. 2016 [48] Case report	1, 65 year old	-	Hysterectomy with bilateral salpingo-oophorectomy bilateral pelvic and para-aortic lymphadenectomy	-	Vimentin + CD99 + p53 + CD56 + CD10 + SMA + PanCK + EMA + CK7 + CK19 + Estrogen receptor (ER) +	Not Performed	UTROSCT	-	12 months	-
38	Umeda et al., Jan 2014 [3] Case series	Case 1: 38 years old Case 2: 57 year old	-	Case 1: Total hysterectomy, bilateral salpingo-oophorectomy, and pelvic lymphadenectomy Case 2: Hysterectomy and bilateral salpingo-oophorectomy	Case 1: left internal iliac lymph node Case 2: -	Case 1: Calretinin + CD99 + CD56 + AE1/AE3 + WT1 + Estrogen receptor (ER) + Progesterone receptor (PR) + MIB-1 + Case 2: Calretinin + CD99 + CD56 + CD10 + WT1 + Alpha-SMA + AE1/AE3 + Inhibin + Estrogen receptor (ER) + Progesterone receptor (PR) + MIB-1 +	Case 1, 2: Gene fusions of JAZF1-SUZ12 (JJAZ1)	Case 1: UTROSCT Case 2: UTROSCT	Case 1: high- dose progesterone therapy Case 2: -	Case 1: 11 months Case 2: 96 months	Case 1: - Case 2: -
39	Vilos et al., Apr. 2018 [49] Case series	Case 1, 52 year old Case 2: 47 year old	Case 1: - Case 2: -	Case 1: Hysterectomy with bilateral salpingo-oophorectomy Case 2: Hysterectomy with bilateral salpingectomy	-	Case 1: Calretinin + Inhibin + CD99 + SMA + Desmin + Case 2: AE1/AE3 + Vimentin + CD99 + Estrogen Recetor (ER) + p16 + SMA + Desmin + Calretinin +	Not Performed	Case 1, 2: UTROSCT	Case 1: - Case 2: -	Case 1: 36 months Case 1: 12 months	Case 1: - Case 2: -
40	Wang et al., 2022 [6] Case report	1, 42 year old	-	Hysterectomy and bilateral salpingectomy	-	Desmin + Smooth-muscle + Actin + WT-1 + D2-40 + CD 99 + Ki-67 increased	No translocation of the JAZF1 gene was detected	UTROSCT	-	2 months	-
41	Wang et al., Mar. 2003 [50] Case report	1, 34 year old	-	Hysterectomy and bilateral salpingo-oophorectomy	-	AE1/AE3/PCK2 + β-catenin + Vimentin + Desmin + SMA + CD99 + Progesterone receptor (PR) + Estrogen receptor (ER) +	Translocations of t(X;6)(p22.3;q23.1) and t(4;18)(q21.1;q21.3)	UTROSCT	-	12 months	-
42	Yin et al., 2022 [51] Case report	1, 51 year old	-	Hysterectomy, bilateral salpingo-oophorectomy, and regional lymph node dissection	-	AE1/AE3+ Cam5.2+ Progesterone receptor (PR)+ Estrogen receptor (ER)+ WT1+ CD56+ Desmin+ TL1+ Calretinin+ CD99+ Synaptophysin+	GREB1–NCOA1 fusion detected	UTROSCT	-	12 months	-
43	Zalewska et al., 2014 [52] Case series	Case 1: 50 year old Case 2: 25 year old Case 3: 51 year old Case 4: 63 year old Case 5: 24 year old Case 6: 64 year old	Case 1: - Case 2: - Case 3: - Case 4: - Case 5: - Case 6: -	Case 1: Subtotal hysterectomy, bilateral salpingo-oophorectomy Case 2: Operative Hysteroscopy Case 3: Subtotal hysterectomy, bilateral salpingo-oophorectomy Case 4: Subtotal hysterectomy, bilateral salpingo-oophorectomy Case 5: Operative Hysteroscopy Case 6: Subtotal hysterectomy, bilateral salpingo-oophorectomy	Case 1: - Case 2: - Case 3: - Case 4: - Case 5: - Case 6: -	Case 1: CD10+, SMA+, Calretinin+, Progesterone Receptor (PR)+, MIB-1+ Case 2: CD10+, SMA+, DES+, Calretinin+, Inhibin+, CKAE1/3+, Progesterone Receptor (PR)+, MIB-1+ Case 3: CD10+, Calretinin,+ Progesterone Receptor (PR)+ Case 4: CD10+, SMA+, DES+, CKAE1/3+, Calretinin+, Inhibin+, CKAE1/3+, Progesterone Receptor (PR)+, MIB-1+ Case 5: CD10+, SMA+, CKAE1/3+, Calretinin+, Inhibin+, Progesterone Receptor (PR)+, MIB-1+ Case 6: CD10+, CKAE1/3+, Calretinin+, Inhibin+, Progesterone Receptor (PR)+, MIB-1+	Not Performed	Case 1, 6: UTROSCT	Case 2, 3, 4, 5: gestagens	Case 1: 174 months Case 2: 84 months Case 3: 66 months Case 4: 60 months Case 5: 54 months Case 6: 36 months	Case 1, 6: -
44	Zhang et al. Dec. 2018 [53] Case series	Case 1: 64 year old Case 2: 33 year old	Case 1: - Case 2: -	Case 1: Hysterectomy, bilateral salpingo-oophorectomy Case 2: Hysterectomy, bilateral salpingectomy, and bilateral ovarian biopsy	Case 1: - Case 2: -	Case 1: Vimentin + Calretinin + WT-1 + Cytokeratin (CK) + Progesterone receptor (PR) + Ki-67 increased Inhibin + CD10 + CA125 + p16 + Case 2: CD99 + SMA + Calretinin + Vimentin + Desmin +	Case 1, 2: Not Performed	Case 1, 2: UTROSCT	Case 1: - Case 2: -	Case 1: 12 months Case 2: 144 months	Case 1: - Case 2: -
45	Zhou, et al., Aug. 2021 [54] Case report	1, 51 year old	-	Hysterectomy and bilateral salpingectomy	-	Ki-67 increased Vimentin + CD99 + CK +	Not Performed	UTROSCT	-	58 months	-
	Total cases: 66	Median age: 49.7 (24–77 year old)	Tamonxifen: 3 APC Gene: 1	H + BSO: 37 H + BS: 10 H + BSO + LND: 12 Hysteroscopy: 5 Myomectomy: 2	Metastasis: - 2 lymph node - 2 ovarian node - 1 cervix			UTROSCT: 66	F-U: 63 RT: 1 CHT: 3 Hormonal Therapy: 2 Not revealed: 1	Median Time: 49.7 months/59 cases Not Revealed: 7	Recurrence: 22% (13/59 cases)

**Table 2:** Site recurrence of UTROSCT.

Local Recurrence	Site	Number of Recurrences
	Ovaries	1
	Pelvic mass	8
	Vaginal vault	1
	Carcinomatosis	2
Distant recurrence		
	Lymph Node	1
	Liver	1
	Bowel	2
	Omentum	3
	Lung	1
